# No Alteration Between Intrinsic Connectivity Networks by a Pilot Study on Localized Exposure to the Fourth-Generation Wireless Communication Signals

**DOI:** 10.3389/fpubh.2021.734370

**Published:** 2022-01-13

**Authors:** Lei Yang, Qingmeng Liu, Yu Zhou, Xing Wang, Tongning Wu, Zhiye Chen

**Affiliations:** ^1^China Academy of Information and Communications Technology, Beijing, China; ^2^Hainan Hospital of Chinese People's Liberation Army General Hospital, Hainan, China

**Keywords:** radiofrequency exposure, long-term evolution, resting-state fMRI, intrinsic connectivity network, dynamic connectivity

## Abstract

Neurophysiological effect of human exposure to radiofrequency signals has attracted considerable attention, which was claimed to have an association with a series of clinical symptoms. A few investigations have been conducted on alteration of brain functions, yet no known research focused on intrinsic connectivity networks, an attribute that may relate to some behavioral functions. To investigate the exposure effect on functional connectivity between intrinsic connectivity networks, we conducted experiments with seventeen participants experiencing localized head exposure to real and sham time-division long-term evolution signal for 30 min. The resting-state functional magnetic resonance imaging data were collected before and after exposure, respectively. Group-level independent component analysis was used to decompose networks of interest. Three states were clustered, which can reflect different cognitive conditions. Dynamic connectivity as well as conventional connectivity between networks per state were computed and followed by paired sample *t*-tests. Results showed that there was no statistical difference in static or dynamic functional network connectivity in both real and sham exposure conditions, and pointed out that the impact of short-term electromagnetic exposure was undetected at the ICNs level. The specific brain parcellations and metrics used in the study may lead to different results on brain modulation.

## Introduction

Wireless communication technology has evolved drastically in the past 20 years. The emergence of the fourth generation (4G) wireless communication technology promoted the widespread applications of mobile network, and vice versa, and 4G rapidly became the popularly used wireless network. The unprecedentedly increasing exposure to radiofrequency (RF) field provoked public anxieties, especially over the effect on neurophysiological function (1). By the end of the third quarter of 2020, although 5G network had already been commercially deployed in many countries for 1 year, there were still 5.82 billion 4G subscriptions (accounting for 62.1% of global subscription) (2). Therefore, it is necessary to investigate the exposure effect of 4G wireless signal.

Subjects who attribute health complaints to everyday levels of electromagnetic fields are suspected of having electromagnetic hypersensitivity, and their symptoms include impaired sense of smell, feeling of pressure in ear, dizziness, and difficulties in concentration (3). However, many studies ascribed the symptoms to psychological suggestion due to the lack of proof of causality (4, 5). The analysis using neuroimaging techniques may help elucidate the concern whether RF radiation exposure would disturb behavioral cognitive function.

The human brain possesses intrinsic connectivity networks (ICNs) relating to underlying neural activity (6). They maintain structural stability at resting state and could be decomposed as spatial-distributed components (independent components, IC) with highly temporal-correlated fluctuations using independent component analysis (ICA) (7) or seed-based analysis (8). Functional network connectivity (FNC), defined by pairwise correlation between ICNs over a certain time course, can measure the averaged connectivity among these ICNs during the scan duration (9). It was conventionally assumed that correlation values stabilized within 4–5 min of data length (7). However, the average over the entire scanning time course may conceal the instantaneous change. Recent research demonstrated that the spontaneous blood oxygen level dependent (BOLD) signals measured during resting state exhibited intrinsic spatiotemporal dynamic organization (10). The dynamic FNC calculated by short time windows was able to track this oscillation over time. Furthermore, the results can be clustered into several connectivity patterns, which may associate with diverse perspectives from unconscious states relevant to anatomical structures to more complex information exchange states (11). By aids of the technique, new breakthroughs have been made in identifying brain dysfunction and cognitive behavior (12, 13). In contrast, relatively few non-ionizing exposure effects have been evaluated in terms of ICNs, and even less on dynamic FNC.

In this work, seventeen healthy participants were recruited and they experienced 30-min exposure. Group-level ICA was performed to decompose ICs across participants from their resting-state fMRI data. We identified 51 ICs in 14 ICNs of interest. Both static FNC over the entire scanning time course and the dynamic FNC using short-time windows were computed. Consequently, these dynamic FNCs were clustered in three states using k-means (14). Statistical analysis was preformed to assess the exposure effect. The study provided a novel approach of understanding the modulation of brain functional connectivity by RF radiation.

## Methods and Materials

### Participants and Experiment Settings

Seventeen healthy right-handed participants including 9 men and 8 women aged 26.1 ± 4.2 (mean ± standard deviation, from 18 to 38) were recruited for this study. They were asked to complete a Medical History Questionnaire before being admitted to the study and none of them had a history of mental illness or disorders related to cognitive dysfunction. They were asked to keep away from caffeine, alcohol, and electronic products the day before experiments. All of them were informed fully of the details and signed a written informed consent. This study conformed to the principles outlined in the Declaration of Helsinki and was approved by the local ethics committee.

**Figure 1:**
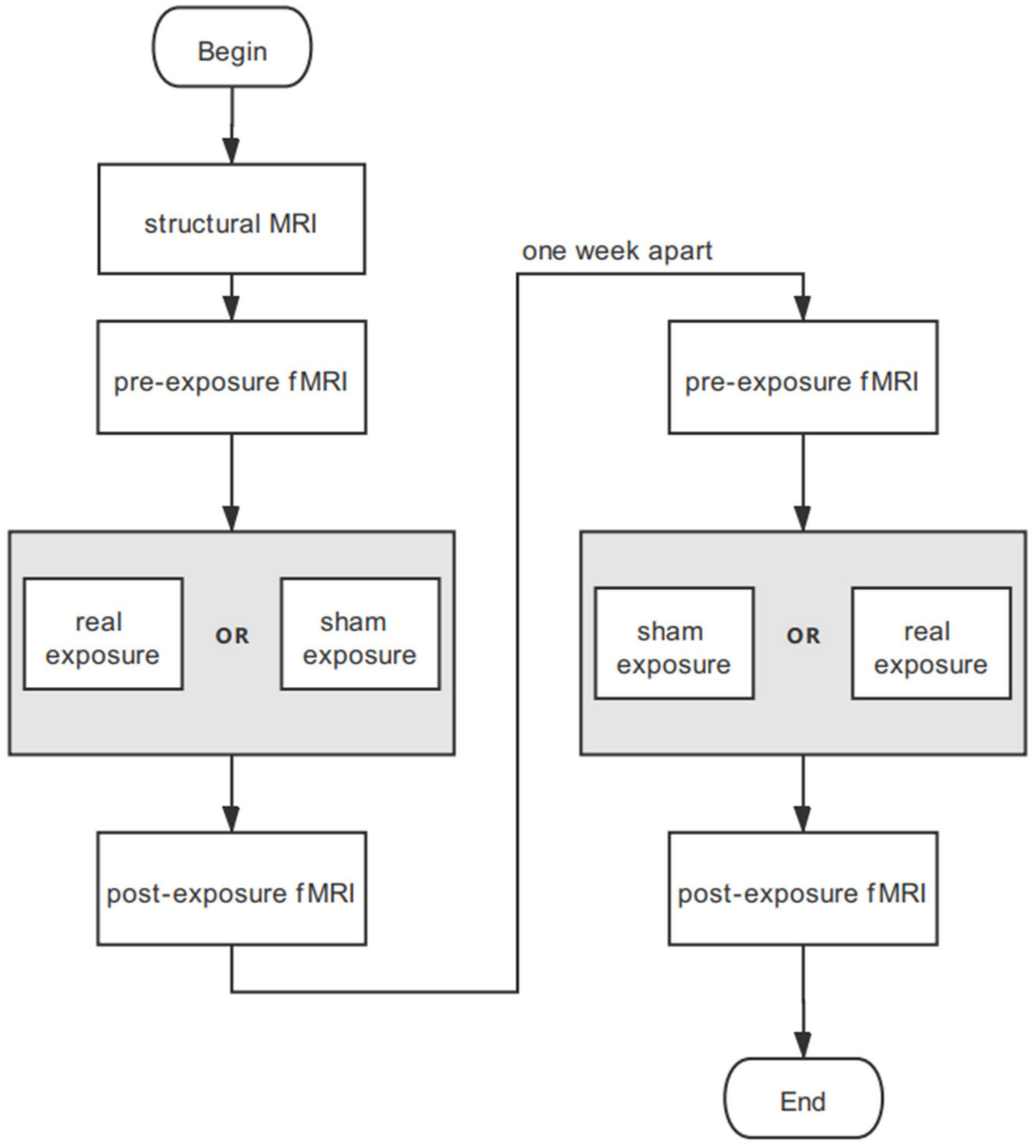
Experimental procedure for each participant. Neither participants nor operators know the exposure sequence.

As the paradigm in Figure 1 shows, the experiment was divided into two sessions with an interval of 1 week. Each session consisted of three stages: fMRI, experiment conditions for 30-min real or sham exposure, and immediate fMRI again. Structural MRI was conducted for participants before the two sessions. The experiment was designed double blind. The real and sham exposure conditions were allocated randomly and counterbalanced across participants. Participants were asked to stay as still as possible. They started scanning immediately after the exposure so that the fMRI data collection could be initiated within 5 min. All participants reported that they kept conscious during the experiments.

**Figure 2:**
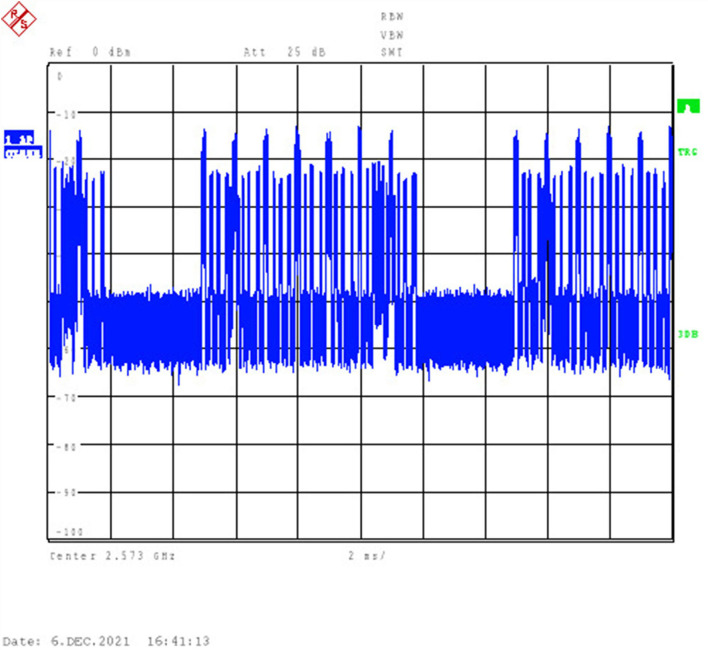
Time-domain signal generated by CMW 500 and visualized by Rohde and Schwarz FSU26 Spectrum Analyzers.

The exposure was carried out in an anechoic chamber to avoid interference from the environment. A signal generator (CMW 500, Rohde and Schwarz, Munich, Germany) was used to generate QPSK modulated time-division LTE signals at 2.573 GHz. The radio frame (total length: 10 ms) consisted of 10 subframes, each 1 ms in length. In the exposure experiment, the maximal emission configuration was opted (simulating 6 uplink subframes in the radio frame). The radiation duration (including the uplink subframes and uplink pilot time slot subframes) accounted for 63.3% of the total frame length, which mimicked the maximum number of the uplink subframes, as prescribed by 3GPP (15). The time domain character is shown in Figure 2.

**Figure 3:**
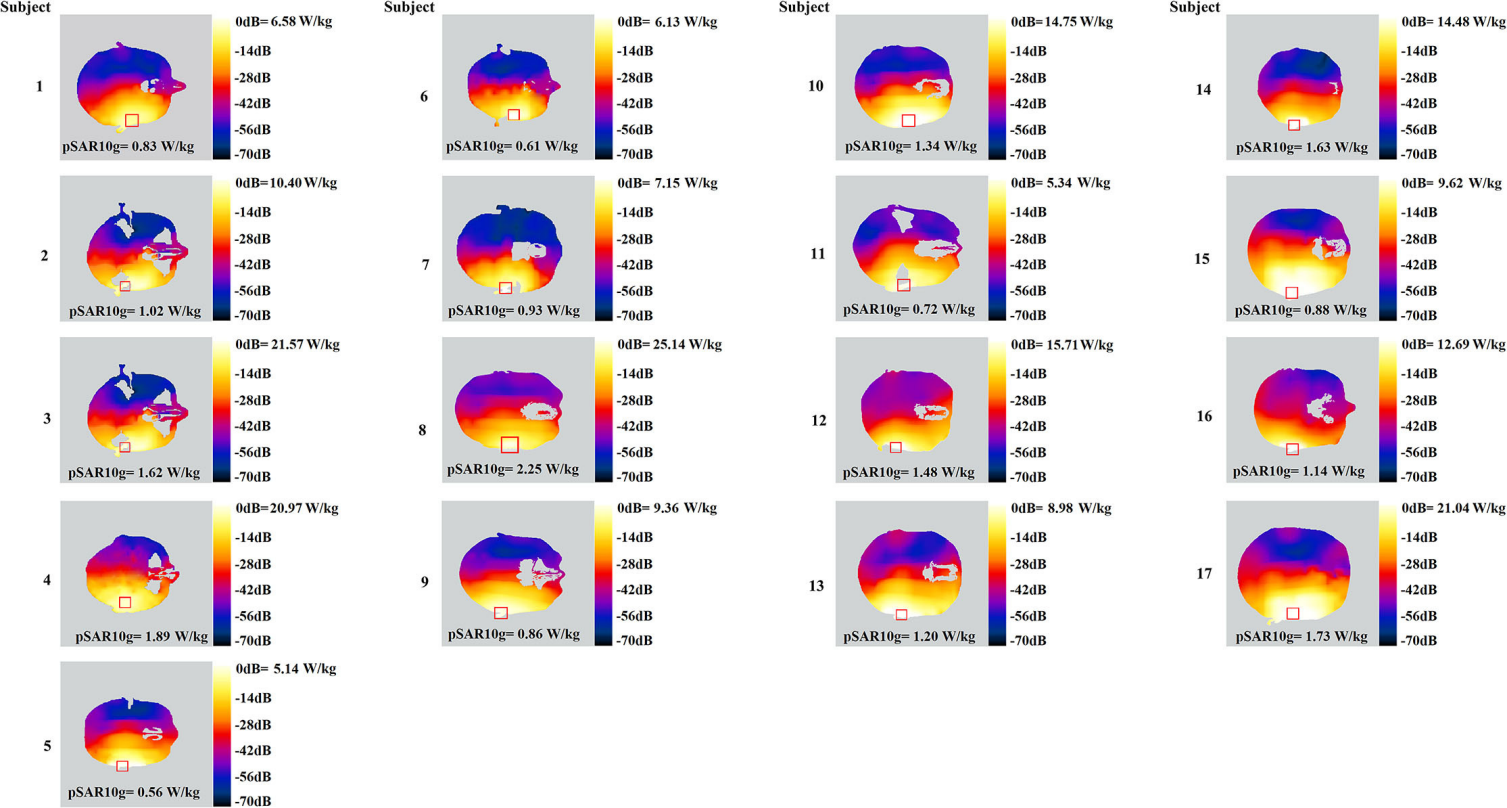
SAR distribution on the transverse slice at the peak value level, for each of the 17 subjects. Square shape delineates the region of pSAR10g on the slice.

Signals were then amplified by an RF power amplifier (AR40S1G4, AR, WA, US). A standard dipole antenna (D2600V2, SPEAG, Zurich, Switzerland) exposed the subjects. Using dipole antenna could avoid the brain activation from temperature rise and sound due to the operation of the mobile phones. The distance between the antenna and the right ear of each participant was stuck to 1 cm. The reflected power due to the existence of the head was monitored and compensated so that the net power output to the dipole antenna was constant (23.0 ± 0.5 dBm). The power distribution in the head was calculated by finite-difference time-domain simulations. The results indicated that the peak SAR averaged over a 10-g mass (pSAR10g) was below 2.00 W/kg for all subjects during real exposure (Figure 3), with a mean ± standard deviation as 1.22 ± 0.24 W/kg. To note, individual head modeling was developed by a semiautomatic segmentation method (16), using in-house tool (17) and iSEG (ZMT, Zurich, Switzerland).

### Data Acquisition and Preprocessing

All the MR data were generated from a 3.0 T system (SIGNA EXCITE, GE Healthcare) with a conventional eight-channel phased array surface coil. The T1-weighted images were acquired at the beginning with repetition time (TR) = 6.8 ms, echo time (TE) = 2.9 ms, field of view = 22 cm, matrix size = 256 × 256. T2-weighted functional images of the whole brain were collected using an echo-planar imaging sequence with TR = 2 s, TE = 30 ms, slice thickness = 3 mm, matrix size = 64 × 64, flip angle = 90°, gap = 0.8 mm. The pre- and post-exposure sessions shared the same parameters, and each lasted for 6 min to collect 180 image volumes. Thirty-two transversal slices were acquired in ascending order for each volume.

Preprocessing was conducted using an automatic pipeline based on SPM 12 (https://www.fil.ion.ucl.ac.uk/spm/software/spm12/). The first ten presteady-state volumes of the EPI time series were firstly removed. Realignment was implemented firstly considering the sequential acquisition. Images were registered to the first image in the series. Then slice timing was performed with the middle one as reference. A single T1-weighted image was co-registered with the corrected average functional image. Then all the functional images were spatially normalized using parameters estimated by nonlinearly registering gray matter and white matter images into MNI space. Their resampled voxel was 3 mm × 3 mm × 3 mm. Finally, the volumes were spatially smoothed with a Gaussian kernel of 6 mm full width at half maximum.

### Group-level ICA to Derive ICNs

Independent component analysis (18), as a data-driven method, is a suitable tool to investigate ICNs at resting state. However, ICA is typically performed separately on each subject, leading to incompatible decompositions across subjects. Consequently, several group-level ICA methods for multisubject analysis have emerged (19).

By the method, the individual voxelwise time-course data were *z*-scored to reduce the variability, followed by a principal component analysis (PCA) to reduce the complexity of the individual data from 170 to 150 volumes using a standard economy-size decomposition. Next, individual data were temporally concatenated as (1):


(1) $$\begin{array}{r} Y\equiv {\left[{Y_{1}}^{\text{T}},\ldots ,\;{Y_{M}}^{\text{T}}\right]}^{\text{T}} \end{array}$$

where, *Y*_*i*_ is *T*_1_-by-V matrix containing the data of subject *i*; $Y\in {\mathbb{R}}^{{\text{T}}_{\text{1}}\text{M}\times \text{V}}$; *T*_1_ is the PCA-reduced time course; *V* is the total number of the voxels for each image; *M* is the number of the dataset to be evaluated.

Consequently, PCA was conducted on the group level to reduce time-course dimension and yielded (2)


(2) $$\begin{array}{r} X=AS \end{array}$$

where, *X* is *T*_2_-by-V concatenated imaging dataset reduced by group-level PCA; A is *T*_2_-by-*T*_2_ mixing matrix; and S is *T*_2_-by-V aggregated spatial map. *T*_2_ was predefined as 100 to achieve a sufficient “functional parcellation” of refined cortical and subcortical components corresponding to the well-known anatomical and functional segmentations (20). The selected number corresponded to the previous studies (21).

The Infomax ICA algorithm (22) was repeated 10 times in ICASSO (23) to derive A and S.

Consequently, back reconstruction can be conducted to derive subject-specific spatial maps and time-course signals by (3) and (4) using least square:


(3) $$\begin{array}{r} S_{i}=\;A^{T}G_{i}Y_{i} \end{array}$$

(4) $$\begin{array}{r} R_{i}=G_{i}A \end{array}$$

where, *G*^*T*^ is the *T*_2_-by-*T*_1_ × *M* reducing matrix.

**Figure 4:**
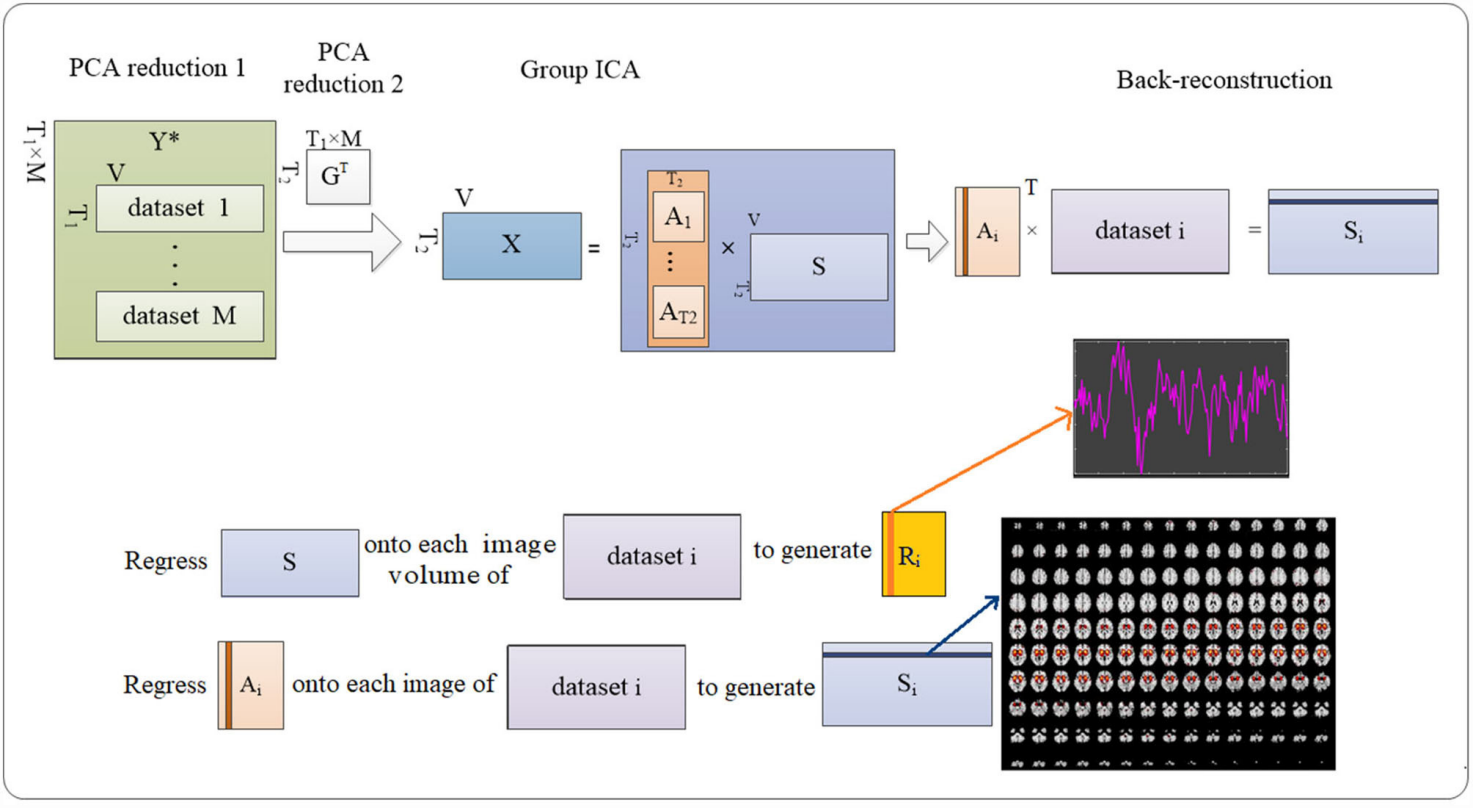
Procedures to detecting the ICs. Group-level ICA decomposes resting-state data from the subjects into ICs (number = 100). Then back reconstruction estimates IC for each subject.

The abovementioned temporal concatenation (24) implemented in the GIFT toolbox (http://mialab.mrn.org/software/gift/) was used in this work for processing functional data from both real and sham exposure conditions. The procedures are described in Figure 4.

The aggregated images of all subject were rewritten in Nifti format so as to enable the labeling for ICs (obtained from spatial maps) according to the RSN templates from the GIFT toolbox (icatb/icatb_templates/RSN.zip).

The generated ICs may contain artifacts and should be removed from analysis according to two criteria. Firstly, the cross correlation between the generated ICs and the RSN templates was calculated, and the ICs with correlation value below 0.2 were considered as artifacts (25). Secondly, the spatial distribution of the IC and its temporal/spectral characteristics were assessed to further screen out the artifacts, and signal ICs should have a high spatial overlap with gray matter and a low overlap with other tissues (26).

### Static and Dynamic FNC

We calculated static FNC over the entire time courses between ICNs. Preprocessing included detrending and low-pass filtering by a fifth-order Butterworth filter with a cut-off frequency of 0.1 Hz (27). Fisher's *z* transformation was then performed.

Consequently, dynamic FNC was computed with the relevant parameters as specified:

- TR = 2 s
- window size: 22 TRs
- step: 1 TR
- number of states: 3 (by *k* means).

**Figure 5:**
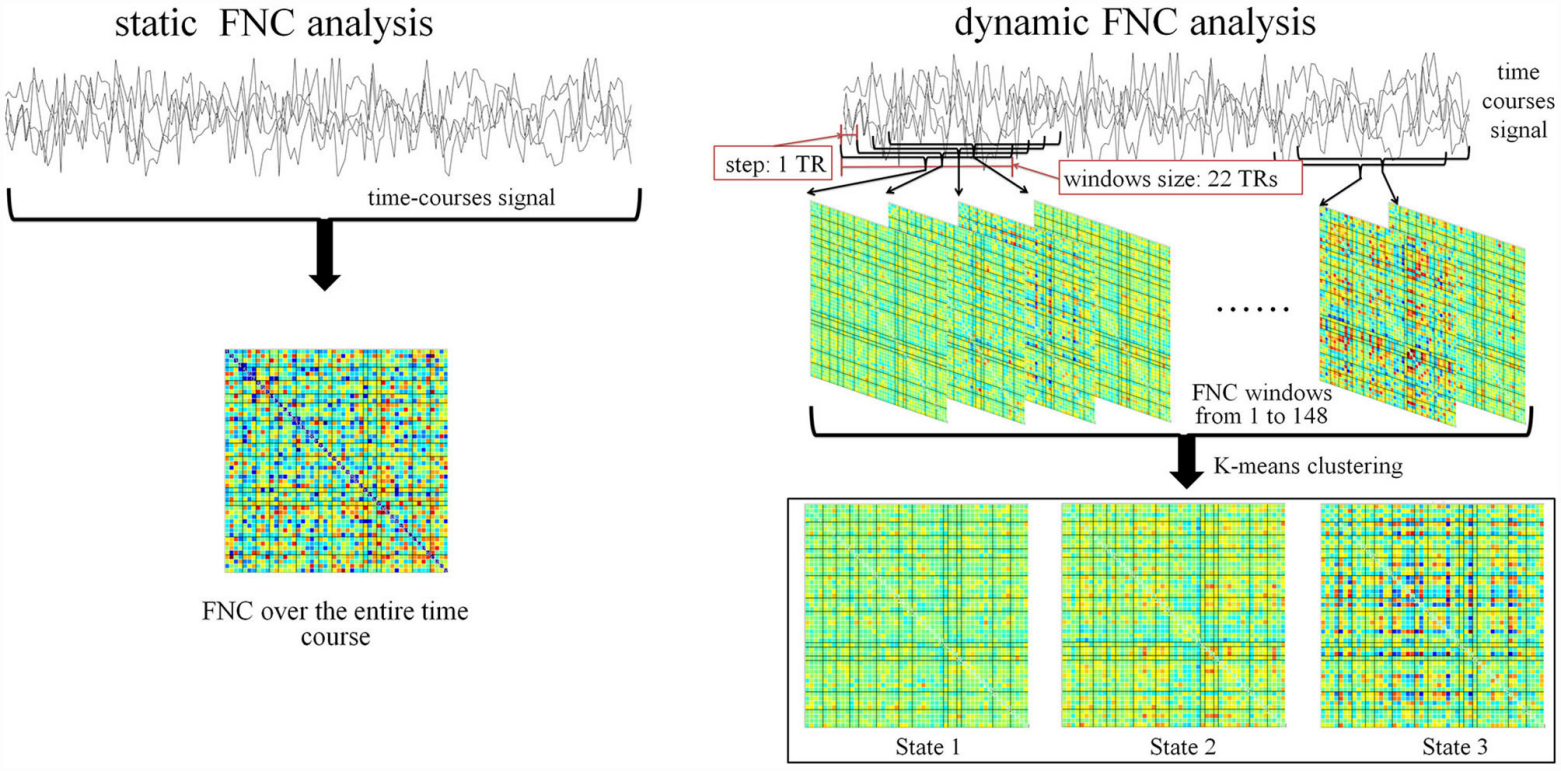
Pipeline for static FNC and dynamic FNC analysis.

To note, TR was set by the imaging protocol. Selection of the window size and step was in accordance to the recommendation by Damaraju et al. (12). Trials have been conducted from 2 to 9 to determine the appropriate number of states. The optimized number was 3 because it ensured that each state contained at least one dataset from qualified subject. The procedure is visualized in Figure 5.

### Statistical Analysis

Paired sample *t*-test was conducted for static FNC within conditions, corrected using FDR with a *p*-value < 0.05. For dynamic analysis, subjects with no less than 10 windows for each state were qualified for statistical comparison.

To investigate if the effects of electromagnetic exposure were driven by certain dynamic FNC states, the median value of all windows for each subject in each state was used for paired sample *t*-test (12). The calculated *p*-value underwent FDR correction with *p* < 0.05.

## Results

**Figure 6:**
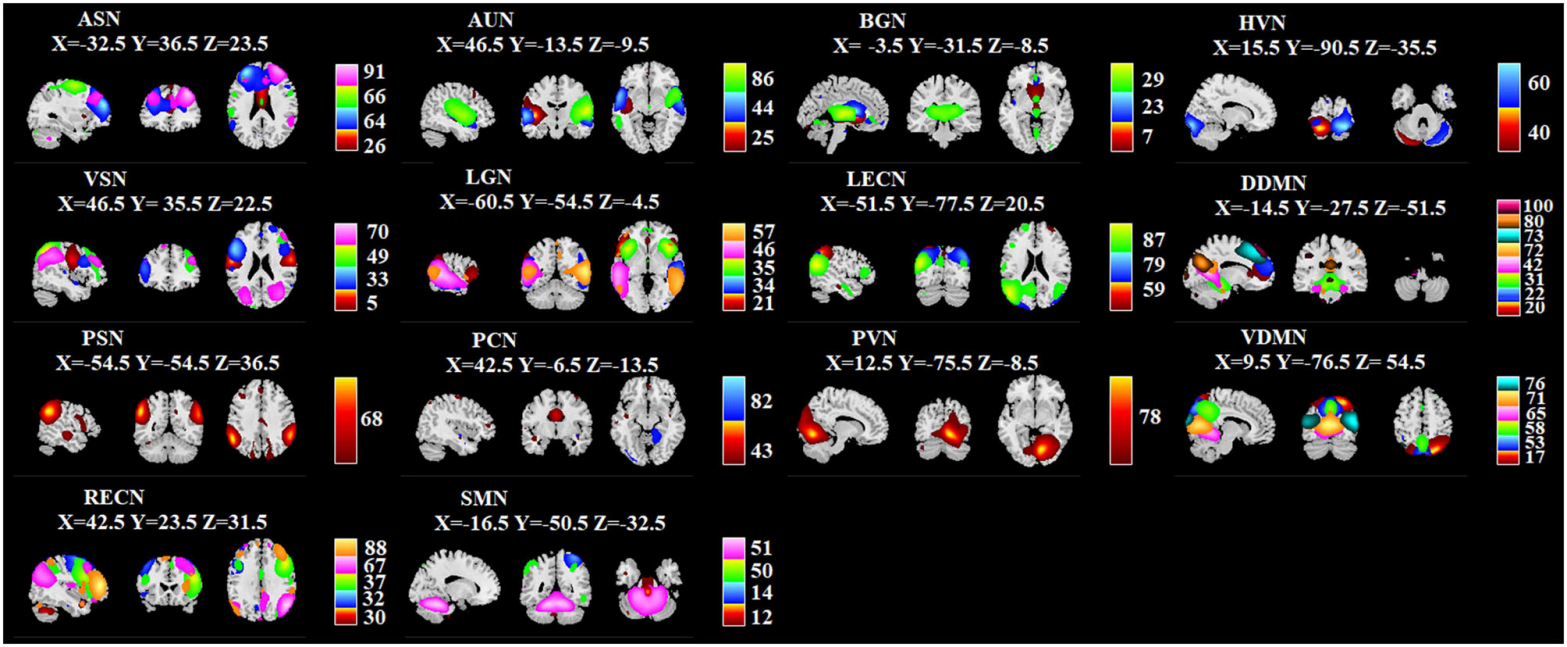
Identified ICs. Within each ICN, the color of the component corresponds to No. of ICs. X, Y, Z corresponds to the MNI coordinates.

Group-level ICA derived 100 spatial ICs was performed to define brain networks, and 100 spatial ICs were generated. By screening out the noise, they were classified into 51 signals whose correlation value was from 0.2058 to 0.5664. Detailed information for ICs are presented in Figure 6: ICs 26, 64, 66, and 91 are anterior salience network (ASN); 25, 44, and 86 are auditory network (AUN); 7, 23, and 29 are basal ganglia network (BGN); 40 and 60 are higher visual network (HVN); 5, 33, 49, and 70 are visuospatial network (VSN); 21, 34, 35, 46, and 57 are language network (LGN), 59, 79, and 87 are left executive control network (LECN); 20, 22, 31, 42, 72, 73, 80, and 100 are dorsal default mode network (DDMN); 68 is posterior salience network (PSN); 43 and 82 are precuneus network (PCN); 78 is primary visual network (PVN); 17, 53, 58, 65, 71, and 76 are ventral default mode network (VDMN); 30, 32, 37, 67, and 88 are right executive control network (RECN); 12, 14, 50, and 51 are sensorimotor network (SMN).

No statistically significant difference was found in static FNC in both real and sham exposure conditions.

**Figure 7:**
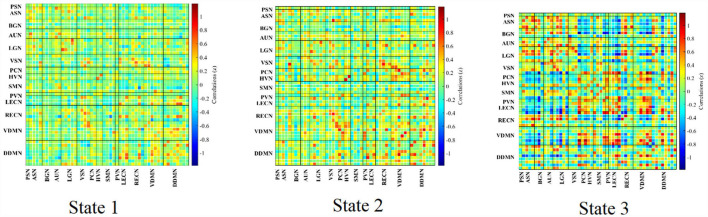
Clustered states in dynamic FNC analysis averaged over the subjects.

The group-specific medians for each state are shown in Figure 7. State 1 accounted for 27% in terms of the occurrence of states across subjects, 66% for State 2, while 7% for State 3. State 1 was similar to State 2, which showed a weak connectivity within each ICN and demonstrated no strong connectivity between ICNs. On the contrary, State 3 showed strong connectivity of ICs within ICN (in particular, with in ASN, AUN, LGN, LECN, VDMN and RECN), enhanced connectivity between VDMN and LECN, PVN, SMN, PCN, PSN, also strong pairwise connectivity among ASN, BGN, AUN, and LGN.

**Figure 8:**
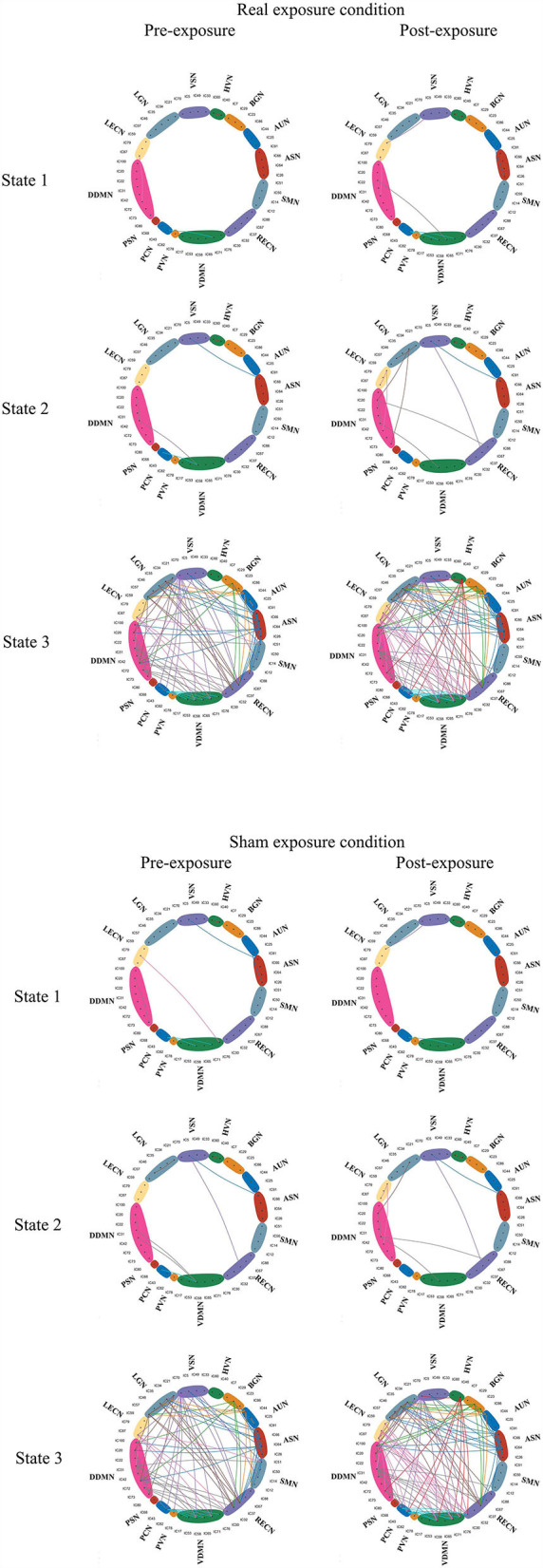
The states derived from the experiments. Connections with correlation coefficients exceeding 0.6 are shown in the Figure (28).

For the dynamic analysis, paired sample *t*-test was performed on the subjects who got at least 10 windows for each state. In pre-exposure of real exposure condition, there were 8 subjects in State 1, 13 subjects in State 2, 3 subjects in State 3. In post-exposure of the same condition, there were 5 subjects in State 1, 12 subjects in State 2, 4 subjects in State 3. In contrast, in pre-exposure of sham exposure condition, there were 6 subjects in State 1, 13 subjects in State 2, and 3 subjects in State 3. The post-exposure in the sham exposure condition revealed 7 subjects in State 1, 12 subjects in State 2, and 4 subjects in State 3. No statistically within-condition significant difference has been detected in terms of real and sham exposure conditions. Figure 8 shows the mean correlation (cross subjects) in all states within real and sham exposure conditions.

The raw data for Figures 7, 8 are provided in Supplementary Tables 1, 2.

## Discussion

In this study, we used a data-driven method (group-level ICA) to identify 51 ICs, which belonged to 14 ICNs, covering the functional areas of vision, hearing, and cognitive control etc. The identified components correlated well with the template and provided a reliable basis for subsequent analysis of static and dynamics FNC during resting state for exposure effects.

Accumulating evidence suggested that static FNC resembled the architecture of brain networks elicited by task-based paradigms (29) and reflected anatomical structure (7). However, the human brain activities were dynamic in nature, and thus, dynamic connectivity analysis was an insight tool to investigate the instantaneous change (30). The enriched information convoyed by the analysis might better reflect the temporally fluctuating brain states compared with static connectivity analysis, as shown in previous studies (31).

K-means clustering was used to identify these reoccurring short-term connectivity patterns, being described as FNC states. FNC state represented the large-scale models of neuronal connectivity that considered the repertoire of functional motifs generated by a given structural architecture (32). Three FC states were confirmed in this study. As shown in Figure 7, 8 and as described in Result, State 1 and State 2 generally showed a weak connectivity, and State 3 showed relatively stronger connectivity. The physiological meaning of the three states could be interpreted. During unconstrained resting-state MRI scan, it was possible for subjects to fall in deliberation or even mind wandering. Therefore, the FNC state representing specific cognitive states unlikely followed similar temporal characters across subjects, expect for the increased likelihood of drowsiness or sleep (32). In this study, State 1 was marked by the disconnection within BGN (thalamocortical neural loop) and the weakening connectivity within DDMN and VDMN. The characteristics were consistent with the features of falling to sleep, such as the reduced thalamocortical connectivity and a breakdown of default-mode connectivity (33, 34). State 3 had a stronger connectivity within DDMN, VDMN, and BGN, respectively. It indicated that this state was close to the awake state. State 2 had connectivity pattern in the BGN similar to State 1 but had a fairly strong connectivity in DDMN and VDMN, which could be regarded as a transitional state from drowsiness to waking state. Moreover, dwelling time in State 1 and 2 accounted for 93% of the total occurrence of all states across subjects. It was also consistent with our analysis on various conscious states during MRI. It revealed that the dynamic estimation/clustering approach had an advantage since it was sensitive to spontaneous state-transition during imaging and supported accessibility of the refined dynamic features of the dataset (12).

No statistically significant difference was found in static or dynamic functional connectivity of ICNs in both real and sham exposure conditions. The finding was seemingly inconsistent to the previous relevant literature on static state analysis. We attributed the difference to the distinctive brain parcellation or the metrics using in these studies. For example, Lv et al. (35) demonstrated that short-term LTE EMF exposure would modulate the interhemispheric homotopic functional connectivity, specifically decreasing amplitude of low frequency fluctuations (ALFF) in resting state around the medial frontal gyrus and the paracentral lobule during the real exposure (36). The study was based on brain anatomy of larger scale (hemisphere). Signals averaged over several ICs were computed for connectivity and may conceal the change in terms of individual IC. Wei et al. (37) detected that acute LTE-EMF exposures modulated both localized intraregional connectivity and interregional connectivity with the other voxels were computed. It evaluated the brain modulation on the level of voxels and without the conception of network structure. Yang et al. (38) indicated that acute LTE exposure modulated both the nodal functional connection and graph-based network properties. Those nodes were defined by AAL-90 template and the connectivity was evaluated for the rest of the nodes (39), which was a parcellation for the entire brain (some changes have been reported at the nodes close to Basal Ganglia). In contrast, our analysis was based on the functional parcellation, aiming to delineate more homogeneous and functionally coherent regions (40). The two parcellations were not completely overlapped. Moreover, the graph-based analysis considered the whole brain as a network to study the ensemble changes of its information transmission efficiency, function integration, network collectivization, and other attributes without paying attention to the special ICN architecture. In fact, ICNs, comprised of various physiological regions (ICs), was coordinated to provide integrative services on behalf of the central nervous system and have emerged as fundamental and organizational elements of human brain architecture. The finding may also indicate that the localized near-field exposure with the power emitted by a mobile phone may impact the regional or interregional BOLD dynamics, but would not affect the specific ICN relating to functional or behavior change. The study, as well as the abovementioned publications, provided useful information to comprehensively understand the change of brain function by EMF exposure.

There were several limitations. Firstly, only 17 subjects were exposed in the experiments. For example, in State 3, only 3–4 subjects were eligible for statistical comparison. In fact, many acute exposure studies on neurophysiological effects have the same problem. For example, Danker-Hopfe et al. (41) conducted a literature review and concluded that 14 out 22 papers on RF exposure and EEG included subjects ≤20. In such a case, the statistical power may only detect the large effects. However, the present study, although with limited number of subjects, has its merit as enriching the knowledge of EMF safety. Based on the work, researchers can continue to optimize the experimental design and to accumulate dataset. Secondly, there were no positive controls in the study. In such a case, it would be difficult to interpret the positive findings if any of them were found (although we did not detect them). Actually, we have considered to include positive control, but it was not allowed by the ethical committee since it may involve high risk, especially when the potential health implication of EMF exposure has not been elucidated. Thirdly, during 3 T MRI scan, the subjects were exposed to the static magnetic field, gradient magnetic field, and the RF fields (128 MHz). The effect may confound the results. Previous reports proposed activation of the sites at the anterior cingulate, the insula, hippocampus, and some parts of nasal gandlia (caudate) following exposure to MRI (42). It was consistent with our research on functional connectivity strength (38). In this work, no statistically significant difference has been reported in the real exposure group. This may be due to the clustering of the network which would smooth the particular activation at the locations. Low-frequency pulsed signals may influence sleep EEG (43, 44). Although the subjects were requested to stay awake during the experiments, completely ruling out the possibility that subjects fell asleep or experienced sleepiness during the MRI scan was difficult. In such a case, the impact should be taken into consideration. Moreover, each subject was only scanned for approximately 6 min (in order to minimize the effect of RF exposure during MRI). Longer scanning times (ideally tens of minutes) will improve the robustness for FC variability estimation.

## Conclusion

We evaluated functional connectivity within and between ICNs identified by group-level ICA. Our results showed that there was no statistically significant different in terms of static and dynamic FNC in both real and sham exposure conditions by exposure to LTE signals. Although previous results show that short-term electromagnetic exposure had an impact on the brain in terms of voxel-wise functional connectivity and graph-theory analysis of functional networks, the results of this work point out that the impact of short-term electromagnetic exposure was insufficient to be detected at the ICNs level. Appropriate metrics for evaluating the brain functional change should be discussed. Further work is needed in the perspective of behavioral change.

## Data Availability Statement

The datasets presented in this article are not readily available because the datasets generated and analyzed during the current study are not publicly available due [Data involves the subject's privacy]. Requests to access the datasets should be directed to Lei Yang, yanglei@caict.ac.cn.

## Ethics Statement

The studies involving human participants were reviewed and approved by Chinese People's Liberation Army General Hospital. The patients/participants provided their written informed consent to participate in this study.

## Author Contributions

TW: conceptualization, supervision, and project administration. LY: methodology, formal analysis, and writing—review and editing. QL: methodology, investigation, and writing—original draft. YZ: validation. XW: data curation. ZC: funding acquisition. All authors have read and agreed to the published version of the manuscript.

## Funding

This research was funded by National Natural Science Foundation of China (No. 61971445).

## Conflict of Interest

The authors declare that the research was conducted in the absence of any commercial or financial relationships that could be construed as a potential conflict of interest.

## Publisher's Note

All claims expressed in this article are solely those of the authors and do not necessarily represent those of their affiliated organizations, or those of the publisher, the editors and the reviewers. Any product that may be evaluated in this article, or claim that may be made by its manufacturer, is not guaranteed or endorsed by the publisher.

## Abbreviations

RF: radiofrequency
ICA: independent component analysis
ICN: intrinsic connectivity network
FNC: functional network connectivity
ASN: Anterior salience network
AUN: Auditory network
BGN: Basal ganglia network
HVN: Higher visual network
VSN: Visuospatial network
LGN: Language network
LECN: Left executive control network
DDMN: Dorsal default mode network
PSN: Posterior salience network
PCN: Precuneus network
PVN: Primary visual network
VDMN: Ventral default mode network
RECN: Right executive control network
SMN: Sensorimotor network
AAL: anatomic automatic labeling
ALFF: Amplitude of Low Frequency Fluctuations.
